# *In Situ* Conformational Changes of the Escherichia coli Serine Chemoreceptor in Different Signaling States

**DOI:** 10.1128/mBio.00973-19

**Published:** 2019-07-02

**Authors:** Wen Yang, C. Keith Cassidy, Peter Ames, Christoph A. Diebolder, Klaus Schulten, Zaida Luthey-Schulten, John S. Parkinson, Ariane Briegel

**Affiliations:** a Institute of Biology, Leiden University, Leiden, The Netherlands; b Department of Biochemistry, University of Oxford, Oxford, United Kingdom; c Department of Physics and Beckman Institute, University of Illinois Urbana-Champaign, Urbana, Illinois, USA; d School of Biological Sciences, University of Utah, Salt Lake City, Utah, USA; e NeCEN, Leiden University, Leiden, The Netherlands; f Department of Chemistry and Center for the Physics of Living Cells, University of Illinois Urbana-Champaign, Urbana, Illinois, USA; University of Washington

**Keywords:** chemoreceptor arrays, chemotaxis, cryo-EM, electron cryotomography, Tsr chemoreceptor

## Abstract

Tsr, the serine chemoreceptor in Escherichia coli, transduces signals from a periplasmic ligand-binding site to its cytoplasmic tip, where it controls the activity of the CheA kinase. To function, Tsr forms trimers of homodimers (TODs), which associate *in vivo* with the CheA kinase and CheW coupling protein. Together, these proteins assemble into extended hexagonal arrays. Here, we use cryo-electron tomography and molecular dynamics simulation to study Tsr in the context of a near-native array, characterizing its signaling-related conformational changes at both the individual dimer and the trimer level. In particular, we show that individual Tsr dimers within a trimer exhibit asymmetric flexibilities that are a function of the signaling state, highlighting the effect of their different protein interactions at the receptor tips. We further reveal that the dimer compactness of the Tsr trimer changes between signaling states, transitioning at the glycine hinge from a compact conformation in the kinase-OFF state to an expanded conformation in the kinase-ON state. Hence, our results support a crucial role for the glycine hinge: to allow the receptor flexibility necessary to achieve different signaling states while also maintaining structural constraints imposed by the membrane and extended array architecture.

## Abstract (precis)

In Escherichia coli, membrane-bound chemoreceptors, the histidine kinase CheA, and coupling protein CheW form highly ordered chemosensory arrays. In core signaling complexes, chemoreceptor trimers of dimers undergo conformational changes, induced by ligand binding and sensory adaptation, which regulate kinase activation. Here, we characterize by cryo-electron tomography the kinase-ON and kinase-OFF conformations of the E. coli serine receptor in its native array context. We found distinctive structural differences between the members of a receptor trimer, which contact different partners in the signaling unit, and structural differences between the ON and OFF signaling complexes. Our results provide new insights into the signaling mechanism of chemoreceptor arrays and suggest an important functional role for a previously postulated flexible region and glycine hinge in the receptor molecule.

## INTRODUCTION

**FIG 1:**
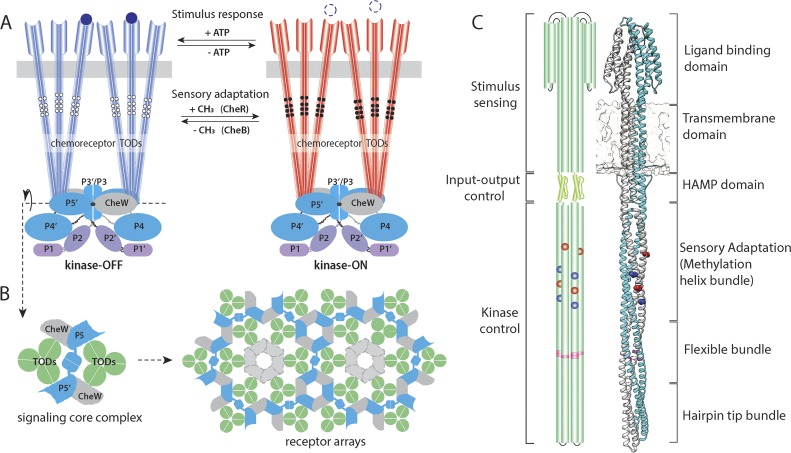
Functional architecture of the E. coli chemoreceptor array and the Tsr receptor. (A) A two-state model of receptor signaling in the core complex, viewed from the side with the cytoplasmic membrane (gray rectangle) near the top. The CheA homodimer and two molecules of CheW bind to the hairpin tips of two receptor trimers. The five CheA domains are designated P1 to P5 in one subunit and P1′ to P5′ in the other. White and black circles indicate the modification states of the receptor methylation sites in the kinase-OFF (white circles, EEEE sites) and kinase-ON (black circles, QQQQ sites) output states. (B) A top-down cross-section through the protein interaction region of the signaling core unit. Core units assemble into an extended receptor array through hexagonal, six-membered P5-CheW and CheW-CheW rings. (C) Cartoon and atomic model of the E. coli serine receptor (Tsr). The Tsr homodimer consists mainly of alpha-helical segments (cylinders, drawn approximately to scale) organized in four-helix bundles. Four methylation sites are indicated in each subunit, with red indicating glutamyl residues (E493 and E304) and blue glutaminyl residues (Q297 and Q311) in the wild-type receptor. A fifth Tsr methylation site (E502) is not shown or discussed in the text because it is less critical for sensory adaptation (67). Glycine residues (G340, G341, and G439), located in the middle of the flexible bundle, comprise the glycine hinge (highlighted in magenta). Atomic model of the full-length Tsr is built based on the structure of HAMP-Tsr fusion (PDB entry 3ZX6) and the ligand binding domain of Tsr (PDB entry 3ATP) (42, 68).

Most motile bacteria sense and track chemical gradients in their environment, a behavior called chemotaxis (1, 2). Chemotactic signaling has been extensively studied in the model organism Escherichia coli and is especially notable for its high sensitivity, signal amplification, and wide dynamic range (3–5). Transmembrane chemoreceptors bind ligands in the periplasm and relay signals across the inner membrane to modulate the autophosphorylation activity of the cytoplasmic histidine kinase CheA (6). Attractant stimuli suppress CheA kinase activity, reducing the flux of phosphoryl groups to the cytoplasmic response regulator CheY. Phospho-CheY binds to the flagellar motor and biases its rotation from the default counterclockwise direction to clockwise. To follow chemoeffector gradients, the chemotaxis system needs to constantly fine-tune its detection sensitivity. Sensory adaptation is made possible by two enzymes: the methyltransferase CheR, which adds methyl groups at specific glutamyl residues in the cytoplasmic portion of chemoreceptors, and the methylesterase CheB, which removes methyl groups from these same sites (7). A fully methylated receptor elicits high CheA activity (i.e., kinase-ON), while a fully demethylated receptor downregulates CheA activity (i.e., kinase-OFF) (Fig. 1A).

E. coli chemoreceptor signaling complexes assemble into extended membrane-bound arrays at the cell pole, which integrate signals from thousands of chemoreceptors through a highly ordered baseplate of CheA kinases and CheW coupling proteins. The signaling core unit comprises two receptor trimers of dimers (TODs), one CheA homodimer, and two CheW proteins (8–10). This minimal functional unit is also the structural core unit in the array (11–13). By linking together at specific interfaces between CheA and CheW, core units form an ∼12-nm-spaced hexagonal array with a receptor trimer at each vertex (Fig. 1B). This hexagonal receptor packing not only is found in E. coli but also is likely universal among bacteria and archaea (39). E. coli contains five different chemoreceptors (Tar, Tsr, Tap, Trg, and Aer) for sensing a variety of chemicals. Due to their similar physical length and high sequence conservation at their cytoplasmic tips, all five chemoreceptors integrate into a single, continuous receptor array (9, 14–16).

Tsr, the serine receptor of E. coli, is a 551-amino-acid protein that spans roughly 31 nm perpendicular to the membrane (12, 17). The receptor homodimers consist of three functional modules that mediate stimulus sensing, input-output control, and kinase control (Fig. 1C) (1, 3). Ligands bind to receptors either directly or indirectly via periplasmic binding proteins (PBPs) at the ligand-binding domain in the periplasm. The signal is then transmitted from the transmembrane domain to the cytoplasmic portion of the receptor through a five-residue control cable that modulates the HAMP (histidine, kinase, adenylate cyclases, methyl-accepting proteins, and phosphatases) domain (18, 19). The 50-residue HAMP domain forms a parallel four-helix bundle that relays stimulus signals to the kinase control module (20, 21), a continuous antiparallel, coiled-coil bundle with a hairpin turn at the membrane-distal end. The methylation helix bundle contains the conserved glutamyl residues that are the sites of adaptational modifications by CheR and CheB. In the flexible bundle (14), three conserved glycine residues reside in a plane transecting the coiled-coil axis, termed the glycine hinge, and may enable the bundle to bend (22, 23). The hairpin tip bundle contains the interfaces through which receptor dimers form trimers as well as directly interact with CheA and CheW (14, 24–26).

Numerous studies have investigated the molecular mechanism of signal transmission in chemoreceptors. A growing body of evidence suggests that kinase activation is achieved through dynamic shifts of local conformational alternations in the contiguous helix regions along the receptor (2, 27). The dynamic-bundle model suggests the kinase-ON output state corresponds to a dynamic, less tightly packed HAMP domain and a stably packed methylation helix (MH) bundle, while the kinase-OFF output state is characterized by a stable HAMP domain and a dynamic MH bundle (28, 29). In addition, the “Yin-Yang model” provides a global view on the long-range allosteric interplay of the kinase control module. Here, the kinase-OFF output state is correlated to a loosely packed MH bundle and a tight packing of the proteins in the interaction region at the hairpin tips. In contrast, the kinase-ON output displays a tight, frozen packing of the MH bundle and a looser helix packing at the tips (30). Together, these studies suggest that sensory signals are propagated along the receptor through dynamic changes in helix bundle packing, which toggle the receptor between the kinase-ON and kinase-OFF output states. The receptor coupling to the kinase is likely assisted by one or more specific residues, which are key to the overall stability of the receptor tips as well as for kinase control through receptor-CheA and receptor-CheW interfaces (31–33).

In this study, we aimed to characterize the signaling conformational changes of Tsr in its near-native cellular context. We combined cryo-electron tomography (cryo-ET) with subtomogram averaging and molecular dynamics simulation to study Tsr in the context of *in vivo*-assembled arrays. Our results show that the compactness of receptor dimers within individual receptor trimers changes with signaling state. In the kinase-ON state, receptors in trimers are more splayed than those in kinase-OFF arrays, a feature that is most distinctive around the location of the glycine hinge. Thus, we propose that the glycine hinge imparts the flexibility necessary for smooth bending in the individual receptors, as well as the changes in compactness at the trimer level. Our results also revealed receptor asymmetry within the trimer that might play a critical role in determining receptor conformational dynamics in the context of the higher-order array lattice.

## RESULTS

### Improved E. coli strains for cryo-ET studies.

To maximize homogeneity of receptor arrays, the strains used in this study contained Tsr as their sole chemoreceptor. In addition, all strains lacked the adaptation enzymes CheR and CheB to maintain the Tsr molecules in a uniform modification state. We imaged three Tsr modification variants: Tsr_QQQQ, which mimics the fully methylated, kinase-ON state; Tsr_EEEE, representing a fully unmethylated, kinase-OFF state; and wild-type Tsr_QEQE, which has an intermediate modification and activity state (3).

The chemoreceptor arrays in E. coli are known to assemble into an ultrastable structure both *in vivo* (34) and *in vitro* (35, 36). This feature has been exploited in previous studies, allowing *in situ* analysis of the assembled array structure in lysed E. coli cells, induced either by a phage lysis gene or antibiotic treatment (13, 34, 37). To increase the size and number of chemoreceptor arrays, previous studies overexpressed array components from plasmids (13, 34). Although array sizes increased substantially, the typical native architecture, especially of the baseplate components (CheA and CheW), seemed to be compromised in such strains (13). To increase array sizes in this study, we imaged strains deleted for *flgM*, in which expression of all class III flagellar and chemotaxis genes is derepressed about 5-fold (38).

### Chemoreceptor arrays maintain native architecture in lysed E. coli.

**FIG 2:**
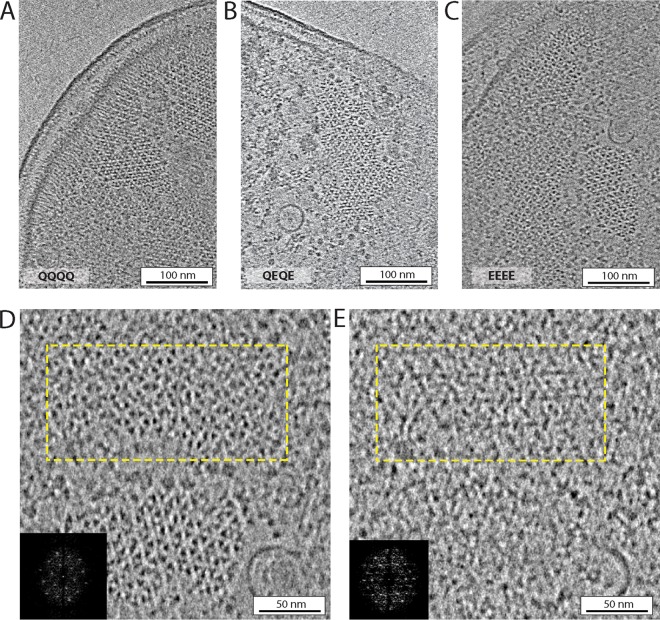
Chemoreceptor arrays imaged by cryo-ET of lysed cells. Panels show 10-nm tomoslices near the cell pole. (A) Tsr_QQQQ. (B) Tsr_QEQE. (C) Tsr_EEEE. (D) Magnified area of a Tsr_EEEE array. (E) Magnified region located 14 nm beneath the array of panel C, showing the ordered CheA distribution in the baseplate. Insets are the power spectra of the regions highlighted by the yellow dashed line in both panels (not to scale). The circular structures in panels B to E are the C-ring of a flagellar motor.

Before preserving specimens by vitrification, we treated E. coli strains at the early exponential growth phase with penicillin G to induce gentle lysis, thereby releasing cytoplasm and flattening the cells. Tomograms of cell poles containing chemoreceptor arrays revealed average cell thickness under 200 nm compared to that of unlysed E. coli cells that are typically more than 500 nm in width (see Fig. S1 in the supplemental material). The receptors retained their well-ordered hexagonal packing, consistent with previous studies (13, 34). However, instead of a single array, we observed several array patches of various sizes, possibly a side effect of lysis treatment on large arrays (Fig. 2A to C). Subtomogram averaging of the receptor hexagons yielded a 12.8-nm regular spacing for arrays in all signaling states. Analysis of the tomographic images also showed that the kinase occupancy at the baseplate was comparable in all strains (Fig. 2D and E). We conclude that all imaged arrays have the expected native architecture.

10.1128/mBio.00973-19.1 FIG S1 The average lysed E. coli cell thickness measured from the tomographic date of samples that contained chemoreceptor arrays. Although in total 30 tomograms were collected for each strain, chemoreceptor complexes for subtomogram averaging were picked from 11, 8, and 7 tomograms for strains expressing Tsr_QQQQ, Tsr_QEQE, and Tsr_EEEE, respectively. Student *t* test (*P* < 0.05) states that cells solely expressing Tsr_QQQQ and Tsr_EEEE are of similar cells thickness after being lysed, while cells expressing Tsr_QEQE were, on average, thinner than those of other groups. Download FIG S1, PDF file, 0.1 MB. Copyright © 2019 Yang et al. 2019 Yang et al. This content is distributed under the terms of the Creative Commons Attribution 4.0 International license.

### Core complex structure in different signaling states.

Subtomogram averages were obtained by receptor-based image alignments and subsequent classification based on the occupancy of CheA underneath the receptor hexagons (Fig. 2D and E). We found two major structural classes: receptor hexagons with three CheA dimers bound at their tips and receptor hexagons lacking CheA. We calculated subtomogram averages for the three-CheA hexagon class for each of the Tsr variants. The coupling protein CheW was poorly resolved in all maps due to its relatively small size (18 kDa) and its preferred orientation in the lysed specimens. Nevertheless, the structural information in the receptor region was only slightly affected by the orientation preference (Fig. S2). We have, therefore, focused this analysis on structural differences between receptors in different output states, in particular highlighting the EEEE and QQQQ maps. Data for Tsr_QEQE can be found in the supplemental material (Fig. S3).

10.1128/mBio.00973-19.2 FIG S2 Simulation of receptor hexagon maps under impact of a missing cone that corresponds to a −60° to 60° tilt scheme. (A) Density map for the receptor hexagon was built with core compel model (PDF entry 3JA6). (B) The map of the receptor hexagon with a soft-edged, missing cone shape mask applied in the Fourier space. From both the side and top view, the missing core has little effect on the size and shape of the receptor; mainly it causes a severe underrepresentation of the CheWs and a shape distortion in the P4 and P5 domains of CheA. Download FIG S2, PDF file, 0.9 MB. Copyright © 2019 Yang et al. 2019 Yang et al. This content is distributed under the terms of the Creative Commons Attribution 4.0 International license.

10.1128/mBio.00973-19.3 FIG S3 Subtomogram averaging of receptor hexagons composed of three dimeric CheAs and different receptor variants. (A) Graphic scheme illustrates the native packing order of the ternary complex in the array lattice, where each hexagon consists of three signaling core units. Components in gray, including the receptor trimers and CheA, are strongly present in the maps, while the coupling protein CheW in the baseplate (white color) is not resolve due to a combination of its low molecular weight and the impact from the missing cone. (B) Overlay of receptor hexagon for strains containing three Tsr receptor variants shows the same native packing order. (C) Averages for three Tsr variants are each low-pass filtered to the same resolution, 25 Å, and present at 1.5-σ level. Download FIG S3, PDF file, 2.2 MB. Copyright © 2019 Yang et al. 2019 Yang et al. This content is distributed under the terms of the Creative Commons Attribution 4.0 International license.

**FIG 3:**
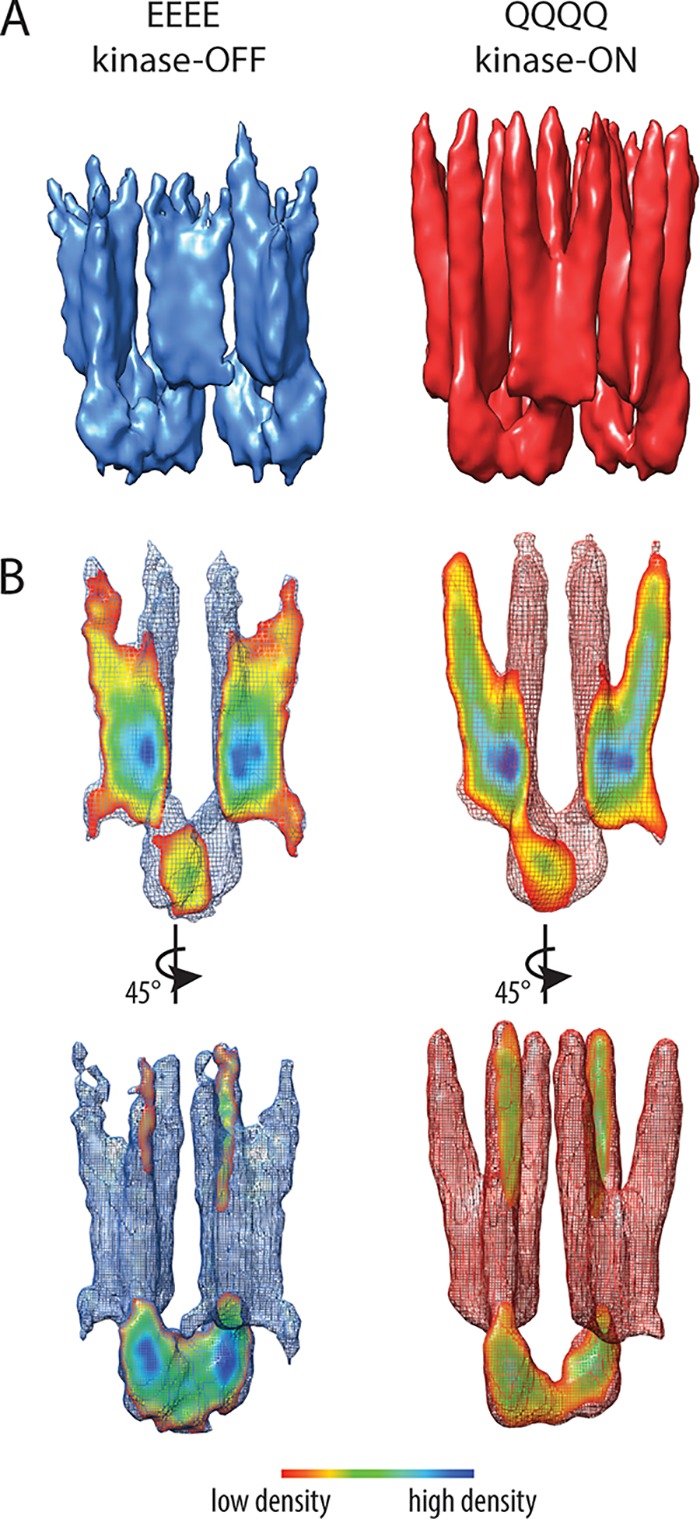
Subtomogram averaging results for Tsr_EEEE and Tsr_QQQQ arrays. (A) Subtomogram average of hexagonal structural units (six Tsr trimers and three CheA dimers). (B) Density maps of chemoreceptor core complexes showing a cross-section through the receptor trimers and the CheA dimer. Mesh surfaces are contoured at 1.5 σ. Cross-section colors indicate the volume density. Red corresponds to a low-density value and blue to a high-density value.

The QQQQ and EEEE receptor hexagon maps were similar in the region near the baseplate (Fig. 3A). In the QQQQ map, the cytoplasmic portion of the receptor from the hairpin tip to just beneath the HAMP domain was clearly visible. In contrast, the HAMP-proximal region of the receptors in the EEEE maps was less well resolved. These results indicate higher structural stability of the receptor trimers near the baseplate in both ON and OFF output states than the membrane-proximal portions of the receptors.

To illustrate state-dependent conformational differences in the core units, we calculated maps for individual core complexes rather than whole hexagons. The resolutions for the core complex maps are 20 Å for QQQQ and 24 Å for EEEE, which are sufficiently similar for tertiary structure comparison (Fig. S4). Alignment of the core complexes helped to improve alignment of the receptor density, especially for the EEEE map (Fig. 3B). A cross-section of the core complex revealed splaying between the receptor dimers in the QQQQ map. The EEEE map also exhibited some receptor splay; however, distinct separation of the individual receptor dimers occurred farther from the hairpin tip. The density distributions of the receptor trimers also exhibited clear differences (Fig. S5). The QQQQ maps exhibited strong receptor density extending nearly to the HAMP domain, whereas the EEEE maps exhibited weaker HAMP-proximal density, implying more structural flexibility.

10.1128/mBio.00973-19.4 FIG S4 Fourier shell correlation (FSC) curves and the map cross-sections of the signaling core unit averaged for all three selected strains. (A) The FSC curves are plotted for each signaling core unit calculated for the corresponding strains. The dashed lines indicate the cutoff at 0.143. All three maps for core units share similar resolutions, which are 20.1 Å, 22.8 Å, and 23.6 Å for Tsr_QQQQ, Tsr_QEQE, and Tsr_EEEE, respectively. (B) The cross-sections of the three maps show the density distribution of the core units. Tsr_QEQE map appears as the intermediate state between the kinase-ON and kinase-OFF biased output states. The surfaces of all maps are rendered at 1.5-σ level in mesh and the cross-section is colored according to the volume density value, where the red color corresponds to a weaker density than the blue. Download FIG S4, PDF file, 0.7 MB. Copyright © 2019 Yang et al. 2019 Yang et al. This content is distributed under the terms of the Creative Commons Attribution 4.0 International license.

10.1128/mBio.00973-19.5 FIG S5 The core unit maps for Tsr_EEEE (blue) and Tsr_QQQQ (red) are low-pass filtered to 25 Å and present at 3-σ level still show a rigid trimer splay in the kinase-ON state and a zipping in the kinase-OFF state. Download FIG S5, PDF file, 0.2 MB. Copyright © 2019 Yang et al. 2019 Yang et al. This content is distributed under the terms of the Creative Commons Attribution 4.0 International license.

The CheA domains P1, P2, and P4 compose a “keel density” protruding beneath the baseplate away from the receptors (34). The size of the keel density appears to be different in the EEEE and QQQQ maps even though the individual CheA domains were not distinguishable (Fig. 3B). The volume of this keel density was 34% greater in the EEEE map than in the QQQQ map. This difference in keel size is consistent with previously reported results for core complexes with different kinase activities (34). The larger keel of CheA in the kinase-OFF state may be due, at least in part, to an unproductive immobilization of the P1 and P2 domains (40).

### Structural differences of receptor trimers.

**FIG 4:**
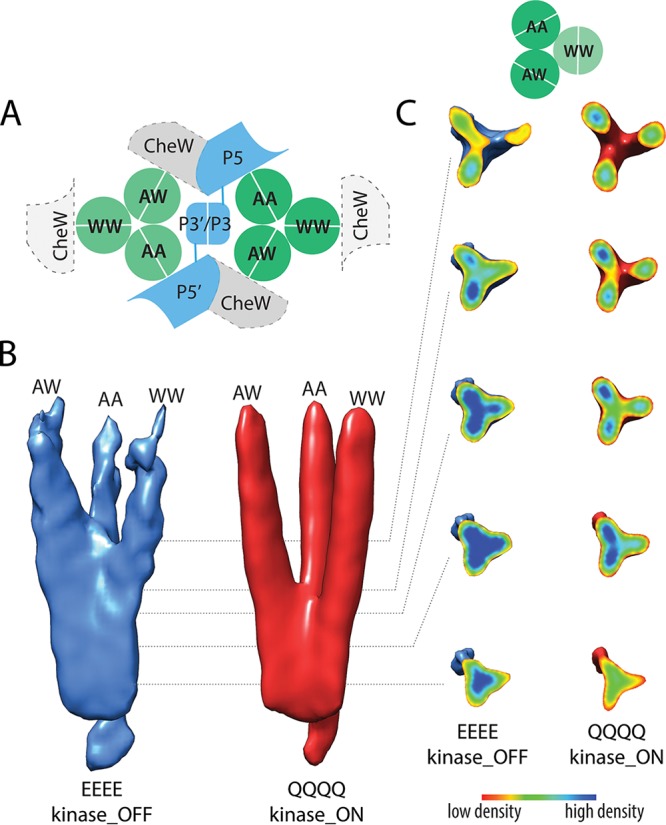
Tsr trimers in different output states. (A) Nomenclature for the three members of a receptor trimer in the signaling core unit. Each Tsr dimer contacts different baseplate components. WW dimers may bind to CheW rings in the array but are shown in light gray with dashed outlines because their extent of CheW occupancy has not yet been established. (B) Density distribution of the receptor trimer of dimers in kinase-ON and kinase-OFF output states. In both states, the AA and AW dimers exhibit a greater coherence than the WW dimer, which exhibited the lowest stability in both maps. (C) The density distribution in different cross-sections of the trimer along the trimer axis, using a color scale from red (low density) to blue (high density).

The analysis of the density maps revealed structural differences not only at the core complex level but also within the receptor trimers. Although receptor dimers interact symmetrically with one another to form a trimer, each receptor molecule also directly interacts with a different component of the base plate (Fig. 4A). Here, we use “AA” to refer to a dimer that interacts with the P5 domain of CheA, “AW” to refer to a dimer that interacts with a CheW bound to CheA•P5, and “WW” to refer to a dimer that interacts with a CheW that has no direct interaction with CheA. We note, however, that in native arrays, not all of the WW dimers may be bound to CheW (41).

The density maps revealed structural asymmetry between the different dimers in a trimer. In the kinase-ON state, the three dimers diverge from the trimer axis at more or less the same location, although the WW dimer diverges a bit closer to the baseplate. The WW dimer also displayed the weakest density of the three dimers. In the kinase-OFF state, the receptors splay farther from the baseplate. We term this observation “dimer zipping” (Fig. 4B) because the dimers form a strong, compact density until they splay apart. After separating from the zipped region, the WW dimer, in particular, shows a decreased density, suggesting a substantial loss of its structural rigidity near the HAMP domain.

The AW and AA dimers also exhibited structural asymmetry in different signaling states. Although a direct, quantitative comparison of the receptor densities in the QQQQ and EEEE maps is challenging, the dimers within each of the maps suggested a subtle structural difference between the AW and AA dimers. In the kinase-ON signaling state, the AA dimer appears to be the most rigid one throughout its full length. In contrast, structural rigidity of the AW dimer seems to extend closer toward the HAMP domain in the kinase-OFF state than in the kinase-ON state. Our data thus reveal signaling-dependent structural or dynamic differences between the members of a receptor trimer of dimers.

### Molecular modeling of the Tsr trimer in different signaling states.

To gain deeper insight into signaling-related changes in Tsr, we investigated the structural differences observed in our cryo-ET data with molecular modeling. We first assigned atomistic structure to the receptor densities seen in our QQQQ and EEEE maps, focusing on a single receptor trimer in each state. Although individual receptor dimers could be clearly distinguished within both maps (Fig. 4), the symmetric nature of the coiled-coil bundles as well as the existence of density corresponding to CheA and CheW prevented the unambiguous docking of lone receptor dimers. Hence, to preserve the known trimer-forming interfaces between receptors during the docking procedure, we first constructed a model of the cytoplasmic portion of the Tsr trimer of dimers (residues 259 to 516) based on existing crystallographic structures (17, 42), using targeted molecular dynamics to reproduce critical interreceptor contacts at the side chain level (Fig. S6). To extract the regions of density corresponding specifically to the receptor trimers within each map, we next docked an existing model of the Thermotoga maritima core signaling complex that contains both CheA and CheW (PDB entry 3JA6) (41). This enabled a reliable interpretation of the baseplate density and consistent positioning of our Tsr trimer model within each map. We then used molecular dynamics flexible fitting (MDFF) simulations (43, 44) to refine the conformational overlap between the receptor trimer model and each map. To ensure the robustness of the obtained fits, a total of five MDFF simulations were conducted for each state, giving rise to nearly identical conformations in each case (backbone root-mean-square deviations of 1.23 ± 0.11 Å for QQQQ and 1.70 ± 0.10 Å for EEEE).

10.1128/mBio.00973-19.6 FIG S6 Atomistic model of the cytoplasmic Tsr TODs (residues 259 to 516) used in this study. Individual monomers within each homodimer are colored in teal and white. Methylation sites (residues Q297, E304, Q311, and E493) and the glycine hinge (residues G340, G431, and G439) are shown with a space-filling representation in yellow and dark grey, respectively. Download FIG S6, PDF file, 0.3 MB. Copyright © 2019 Yang et al. 2019 Yang et al. This content is distributed under the terms of the Creative Commons Attribution 4.0 International license.

**FIG 5:**
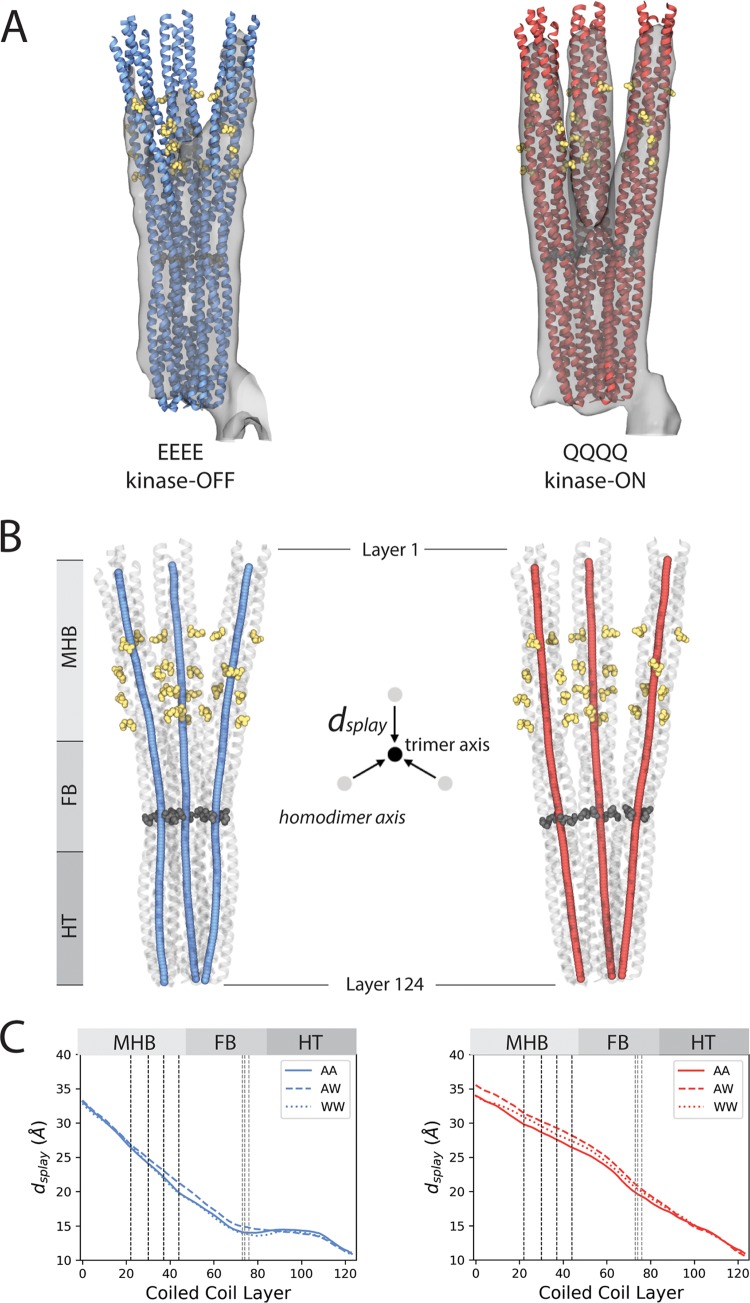
Signal state differences in trimer compactness. (A) Overlays of the EEEE and QQQQ density maps and representative MDFF-derived Tsr backbone configurations. (B) Central axes of the homodimers in each trimer conformation. Receptor regions are the methylation helix bundle (MHB) and modification sites (yellow atoms), the flexible bundle (FB), glycine hinge (dark gray atoms), and the hairpin tip (HT). (C) Plots of the distance between the central axis of each homodimer and the symmetr*y* axis of the trimer (d_splay_). Gray dashed lines denote coiled-coil layers containing the glycine hinge; black dashed lines denote layers containing methylation sites.

Visual inspection of the flexibly fit conformations confirm that the Tsr trimer is markedly more compact, on average, in the kinase-OFF state than the kinase-ON state (Fig. 5A and Movie S1). To quantify this difference, we decomposed the receptor homodimers from the EEEE and QQQQ trimer models into layers based on coiled-coil packing and computed their central axis along with the symmetry axis of the receptor trimer using TWISTER (Fig. 5B) (45). The layer-by-layer distances between the central axis of each homodimer and the trimer axis reveal a considerable inhomogeneity in the overall splay of the kinase-OFF trimer (Fig. 5C). Specifically, whereas the receptors diverge uniformly from the trimer axis in the kinase-ON state, remaining relatively straight and interacting only at the hairpin tip, they exhibit a pronounced bend in the kinase-OFF state that is centered on the glycine hinge. This bending facilitates the transition from a compact trimer configuration, in which the flexible bundle regions of the homodimers interact to one in which they are well separated in the methylation helix bundle region (Movie S1). Similarly, the comparison of the AA, AW, and WW homodimer axes between states highlights that the overall greatest change in each receptor occurs in the flexible bundle region, with the WW homodimer showing the largest difference of the three (Fig. S7). Thus, our simulations provide new molecular insight into Tsr signaling, highlighting, in particular, the key role of the glycine hinge in facilitating the transition between signaling states at the receptor trimer level.

10.1128/mBio.00973-19.7 FIG S7 (A) Overlay between the central axes of representative EEEE (blue) and QQQQ (red) receptor trimer conformations. The distance (d_splay_) between each receptor homodimer and the central axis varies for EEEE and QQQQ (Δd). (B) Plot depicting the distance changes (Δd) of the AA, AW, and WW homodimers shown in panel A, which reflects the inhomogeneity of the trimer compactness changes. Download FIG S7, PDF file, 0.3 MB. Copyright © 2019 Yang et al. 2019 Yang et al. This content is distributed under the terms of the Creative Commons Attribution 4.0 International license.

10.1128/mBio.00973-19.10 MOVIE S1 Linear interpolation between the QQQQ and EEEE receptor trimer conformations. Overlays with each map illustrate the fits derived via MDFF. Color scheme taken from Fig. S6. Download Movie S1, MOV file, 12.3 MB. Copyright © 2019 Yang et al. 2019 Yang et al. This content is distributed under the terms of the Creative Commons Attribution 4.0 International license.

## DISCUSSION

### Signal state affects stability of the methylation helix bundle.

The present study and numerous previous cryo-ET attempts failed to unveil the structure of full-length chemoreceptors *in situ* (11, 12, 34). Although we were not able to resolve the periplasmic, transmembrane, and HAMP regions of the receptors, we were able to clearly show signaling-related conformational differences throughout the kinase control module of Tsr. A particularly distinctive difference was observed in the MH bundle, where the receptors exhibited a more continuous density distribution in the kinase-ON output state than the kinase-OFF state. This observation agrees with the idea that receptor methylation enhances helix-packing interactions (30, 46). Thus, in our cryo-ET results, receptor density in the MH bundle region was less prominent in the EEEE trimers, suggesting that the adaptation region is structurally more dynamic in the kinase-OFF state. It is worth mentioning that conformational heterogeneity in the kinase-OFF data set, due to the aforementioned zipping behavior within trimers, may also contribute to the poorly resolved nature of the MH bundle in this state.

### Role of the glycine hinge in signal state switching.

We suggest that the state-dependent splayed versus zipped arrangements of receptors in the flexible bundle region reflect conformational coupling between the MH bundle and the hairpin tip bundle. To our knowledge, these state-dependent structural differences have not been previously observed in imaging studies. Our MDFF analyses show that the flexible bundle region of the receptor exhibits the most dramatic structural differences between signaling states. Glycine residues, located at the center of the flexible bundle region, likely facilitate splaying in the receptor trimer. The functional role of the glycine hinge in chemoreceptor signaling has been a topic of much speculation and is somewhat controversial. Mutational analyses have shown that side chain replacements at the glycine hinge residues impair or abrogate chemotaxis ability (22, 47). Moreover, several studies have suggested that the glycine hinge introduce structural flexibility to the helix bundle, perhaps to allow bending (14, 22). However, previous MD simulations suggested that the glycine hinge did not show a particularly high propensity to bend in receptors out of the array context (31, 48).

A recent electron microscopy (EM) study of Tar dimers inserted in nanodiscs showed that receptors bent in two areas under these conditions: just below the HAMP domain and around the glycine hinge (23). That study proposed that bending at the glycine hinge was not related to output state but instead was crucial for facilitating receptor clustering without structural clashes. However, that study lacked the structural context of extended arrays, where interactions with CheA and CheW might have substantial effects on receptor structure. Our data indicate that the glycine hinge plays a crucial role in facilitating the dimer-zipping motions required to mediate the conformational shift between kinase-ON and kinase-OFF output states. Bending at the glycine hinge might, for example, serve to structurally couple changes in helix packing of the MH bundle to signaling changes at the receptor hairpin tip. Further improvements in EM maps to subnanometer resolution should elucidate the mechanism of signal propagation through the glycine hinge.

### Stability of the receptor tip in different signaling states.

The Yin-Yang hypothesis proposed that dynamic motions of the MH bundle and the protein interaction region at the receptor’s hairpin tip are coupled in opposition (30). Thus, this model predicts that receptor tips might be “frozen” in the OFF state and relatively “molten” in the ON state. Given that the Tsr protein interaction regions appeared to be quite similar in our kinase-ON and kinase-OFF maps, our cryo-ET data do not support a large dynamic structural difference between the two output states. The tip bundle contains multiple interaction surfaces that maintain the structural integrity of the trimers and the core units. Thus, it seems likely that the tip adopts alternative, stable conformations in both signaling states through structural changes that are small in magnitude. MD simulations of a Tsr dimer proposed a conformational switch at the receptor tip through state-dependent flips in phenylalanine stacking (31). Symmetric rotations of the dimers about the trimer axis could produce those conformational changes at both the Tsr•P5 and Tsr•CheW interfaces (25). Taking these considerations into account, it is plausible that dimer zipping promotes a reversible twisting motion of receptors at the tip region that triggers a discrete conformational switching between signaling output states.

### Effects of receptor signaling state on the kinase.

Receptor signaling state influences the mobility of the CheA P1 and P2 domains in core complexes (34). Our data support this conclusion, because we found that CheA had a larger keel volume in the kinase-OFF state. In addition, we observed dimer zipping in the baseplate region of receptors in kinase-OFF signaling complexes. Thus, it is plausible that conformational coupling between zipped receptors freezes their tightly packed hairpin tips and CheA domains in the kinase-OFF conformational state. In the kinase-ON state, the CheA keel (domains P1, P2, and possibly P4) is less prominent, consistent with a broader range of domain motions. However, we saw no evidence for enhanced mobility of the receptor tips in kinase-ON signaling complexes. We suggest, therefore, that in the kinase-ON state, receptor tips adopt a discrete, structurally stable conformation that frees up CheA domain motions to promote the autophosphorylation reaction. CheA control probably occurs through the receptor/CheW and CheW/CheA.P5 interfaces (25, 32, 33), in turn modulating the CheA•P4 domain (49–51) and possibly the CheA P3/P3′ dimer interface (52).

Our data show that the AW dimer undergoes a change in rigidity between the kinase-ON and -OFF state. Asymmetric signaling within receptor trimers has been previously suggested based on the observation that only one dimer within a receptor trimer conveys ligand-binding information to CheA (52). Our results suggest that conformational changes caused by adaptational modification of individual dimers manifest themselves at the level of receptor trimers to modulate kinase control. Thus, although receptor dimers within a trimer undergo asymmetric conformational dynamics depending on their position within signaling complexes, all three dimers play a role in conveying signals to the kinase.

### Summary.

Despite considerable effort, a complete understanding of the signal transduction events occurring between ligand binding and the regulation of CheA autophosphorylation is still lacking. This can be attributed to the structural complexity of the intact array system, together with the difficulty of analyzing signal transduction events in this context. Our study reveals the conformation dynamics of the E. coli Tsr in its native structural context, highlighting global changes in receptor conformation in different signaling states. Our new observations surrounding (i) stability changes in the methylation helix bundle, (ii) zipping in the flexible bundle region, and (iii) asymmetric rigidity changes at the receptor tips collectively reflect that the conformational changes corresponding to signaling states take place in the whole kinase control module of the receptor rather than a single region. Altogether, our results provide crucial insights into the structural and functional changes in the receptors in the context of native arrays.

## MATERIALS AND METHODS

### E. coli strains.

E. coli strains used in this study are derivatives of RP437, a wild-type chemotaxis derivative of E. coli K-12 (53). The strains were previously described (34) and were further modified by introducing an *flgM* deletion to enhance expression of class III flagellar and chemotaxis genes (see Table S1 in the supplemental material).

10.1128/mBio.00973-19.8 TABLE S1 E. coli strains used in this study. Download Table S1, PDF file, 0.05 MB. Copyright © 2019 Yang et al. 2019 Yang et al. This content is distributed under the terms of the Creative Commons Attribution 4.0 International license.

### E. coli cell lysis and cryo-ET specimen preparation.

E. coli strains were cultured in Tryptone broth at 30°C with 200 rpm shaking overnight. An overnight culture of E. coli was diluted into 50 ml at a 1:100 ratio. The diluted culture was then allowed to grow until its optical density at 600 nm (OD_600_) reached 0.2. Penicillin G potassium salt (Carl Ruth, Karlsruhe, Germany) then was added to the culture for a working concentration of 2,000 UI/ml. After 30 min of incubation at 30°C, the cells from 1 ml culture were collected by centrifugation at 13,000 rpm in a 1.5-ml Eppendorf tube. The supernatant was discarded, and pellets were resuspended in 10 μl phosphate-buffered saline and kept on ice.

The protein A-treated 10-nm colloidal gold solution (Cell Microscopy Core, Utrecht University, Utrecht, The Netherlands) was mixed with penicillin-treated cells at a 1:10 ratio. After brief vortexing, a 3-μl aliquot of mixture was applied to a freshly plasma-cleaned R2/2, 200-mesh copper Quantifoil grid (Quantifoil Micro Tools GmbH, Jena, Germany) and applied to the EM grid in the climate chamber of a Leica EMGP (Leica Microsystems, Wetzlar, Germany). The grid was blotted for 1 s from the carbon-facing side of the grid at room temperature (20°C) and 95% humidity. Plunge freeing was carried out in liquid ethane at –183°C. Grids were stored in liquid nitrogen until data acquisition.

### Cryo-electron tomography.

Data acquisition was performed on a Titan Krios transmission electron microscope (Thermo Fisher Scientific [formerly FEI], Hillsboro, OR, USA) operating at 300 kV. Images were recorded with a Gatan K2 Summit direct electron detector (Gatan, Pleasanton, CA) equipped with a GIF-quantum energy filter (Gatan, Pleasanton, CA) operating with a slit width of 20 eV. Images were taken at a nominal magnification of 42,000×, which corresponded to a pixel size of 3.5 Å. The UCSFtomo software package was used for data acquisition with low-dose mode and dose fractionation within a cumulative exposure of 80 e^−^/Å^2^ (54). All tilt series were collected using a bidirectional tilt scheme which started from 0° to −60° and continued from 0° to 60° tilting with a 2° increment. Defocus was set to −8 μm. A total of 28 tilt series were collected for each strain.

### Tomogram reconstruction and subtomogram averaging.

IMOD software was used for drift correction and bead tracking-based tilt series alignment (55, 56). CTF estimation and correction were done with CTFPLOTTER and CTFPHASEFLIP implemented in IMOD (57). Tomograms were reconstructed for each tilt series by weighted back projection, both with and without a simultaneous iterative reconstruction technique (SIRT)-like filter equivalent to 9 SIRT iterations. Tomograms reconstructed with the SIRT-like filter provided strong contrast for evaluating array distribution, particle picking, and initial template building, while tomograms built by weighted backprojection were used for subtomogram extraction, alignment, and averaging.

Subtomogram averaging was done with the Dynamo software package (58–60). The initial subtomograms were defined as six trimmers of receptor dimers packed in hexagonal order. Subtomograms were manually picked from selected tomograms binned by 2. After coarse alignment based largely on the receptor hexagons, principal component analysis and *k*-mean-based classification was performed based on the CheA occupancy beneath the receptor hexagon. Subtomograms were extracted from tomograms reconstructed by SIRT-like weighted backprojection, since they provided strong contrast for receptor hexagon alignment and CheA-based classification. Each CheA-filled hexagon consists of three signaling core units following C3 symmetry. Subboxing was carried out to extract the individual core units for further alignment. In addition, an extra round of subboxing was done to extract two receptor trimers of dimers from each core unit. For trimer alignment, a soft cylindrical mask was applied to enclose the trimer density. All final maps were calculated from weighted back-projection tomograms. The Fourier shell correlation curves for the core unit maps were calculated with the EMAN2 software package (61). Surface visualization was done using the Chimera software package (62–64). The particle numbers of averages used are listed in the supplemental material (Table S2).

10.1128/mBio.00973-19.9 TABLE S2 The particle numbers for calculating the subtomogram averaging results for the Tsr receptor hexagons with triple CheAs, the signaling core unit, and the Tsr trimers. Download Table S2, PDF file, 0.04 MB. Copyright © 2019 Yang et al. 2019 Yang et al. This content is distributed under the terms of the Creative Commons Attribution 4.0 International license.

### Model building.

A preliminary model of the Tsr trimer of dimers was constructed by aligning a copy of PDB entry 3ZX6 (42), which contains the complete cytoplasmic antiparallel coiled-coil domain of Tsr (residues 259 to 516), with the protein interaction region of each partial homodimer seen in the crystal structure of Tsr trimers of dimers (PDB entry 1QU7, residues 340 to 440). Using VMD, the model was then hydrated with TIP3P water molecules and subsequently neutralized and ionized with potassium and chloride ions to a concentration of 150 mM, resulting in a system containing 239,688 atoms. The complete system was then subjected to an energy minimization followed by a 50-ns equilibration simulation in which the protein backbone was harmonically constrained. Targeted molecular dynamics was then used to reproduce the interhomodimer interfaces seen in PDB entry 1QU7 by minimizing the root mean squared deviation between the backbone and side chain positions in the protein interaction region of the two structures. The resulting model was used as the input structure for subsequent MDFF simulations.

### Molecular dynamics simulations.

All molecular dynamics simulations were carried out using NAMD 2.12 (65) and the CHARMM36 force field (66). MDFF simulations were performed in the NVT ensemble at 310 K for 20 ns. A scaling factor of 0.15 was used to couple backbone atoms to the MDFF potential. Additional harmonic restraints were applied during fitting to prevent loss of secondary structure. Isothermal conditions were maintained by a Langevin thermostat. The r-RESPA integrator scheme with an integration time step of 2 fs was used along with SHAKE constraints on all hydrogen atoms. Short-range, nonbonded interactions were calculated every 2 fs with a cutoff of 12 Å, while long-range electrostatics were evaluated every 6 fs using the particle-mesh-Ewald (PME) method with a grid size of 1 Å.

### Accession numbers.

The EMDB accession numbers for the subtomogram averages of signaling core units at different kinase activation levels reported in this study are EMD-4991 (Tsr_EEEE), EMD-4992 (Tsr_QQQQ), and EMD-4993 (Tsr_QEQE).
