# Supplementary figures and images for: In Situ Conformational Changes of the Escherichia coli Serine Chemoreceptor in Different Signaling States
Source: mBio. 2019 Jul 2;10(4):e00973-19. doi: 10.1128/mBio.00973-19 (PMC6606802; doi:10.1128/mBio.00973-19)

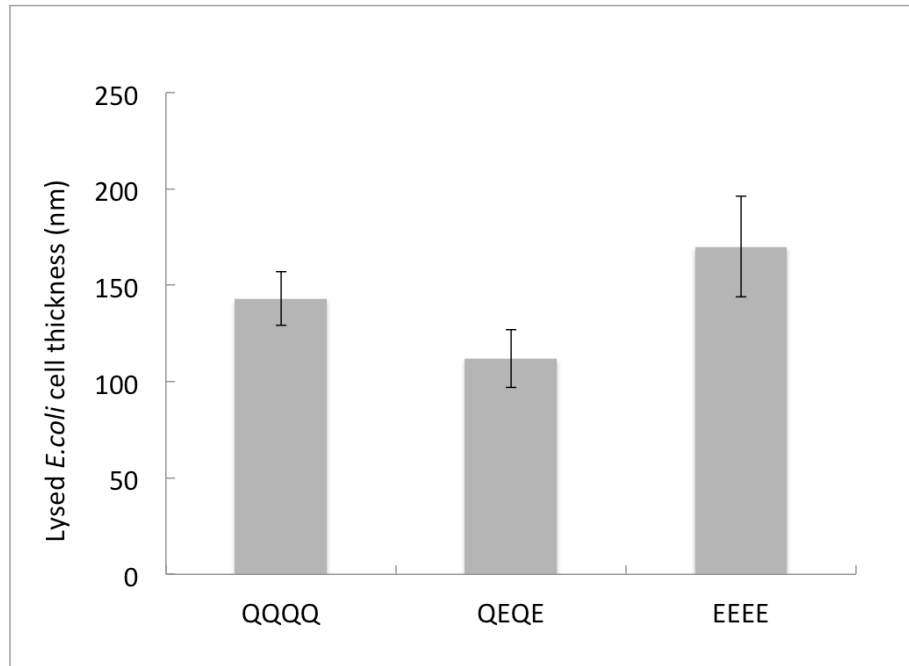

Supplement: FIG S1 [file mBio.00973-19-sf001.pdf]

A

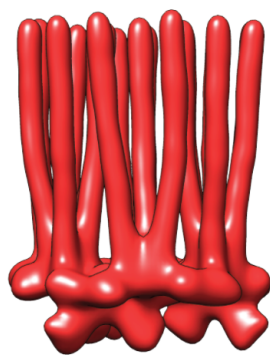

90°

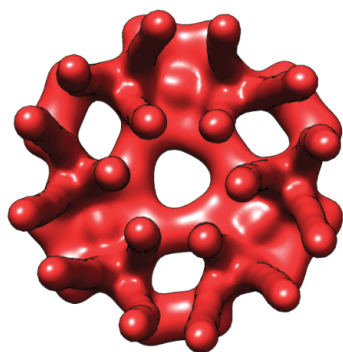

B

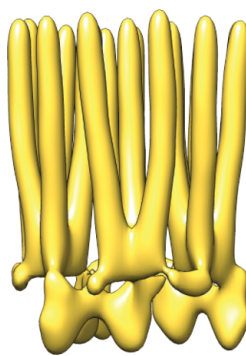

90°

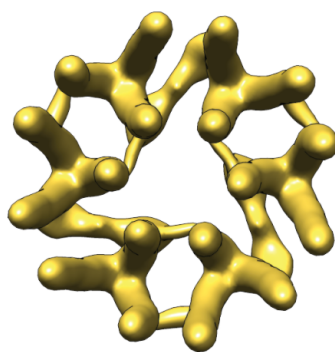

Supplement: FIG S2 [file mBio.00973-19-sf002.pdf]

A

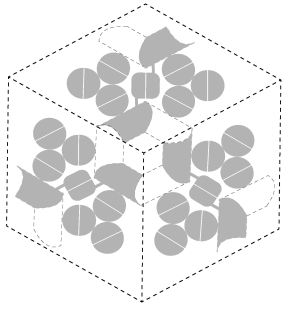

B

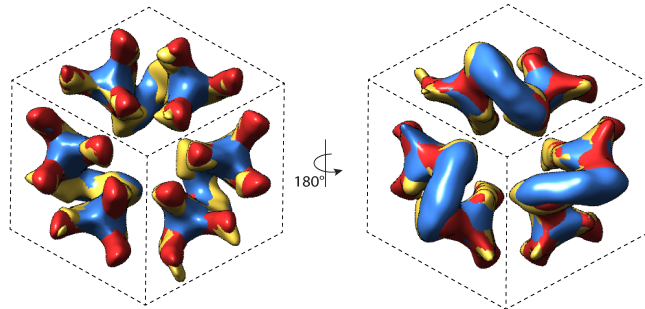

C

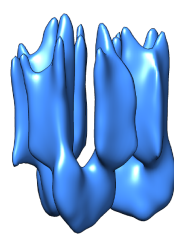

Tsr\_EEEE

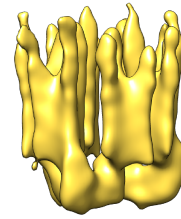

Tsr\_QEQE

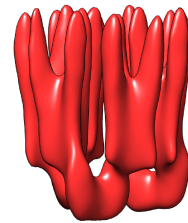

Tsr\_QQQQ

Supplement: FIG S3 [file mBio.00973-19-sf003.pdf]

A

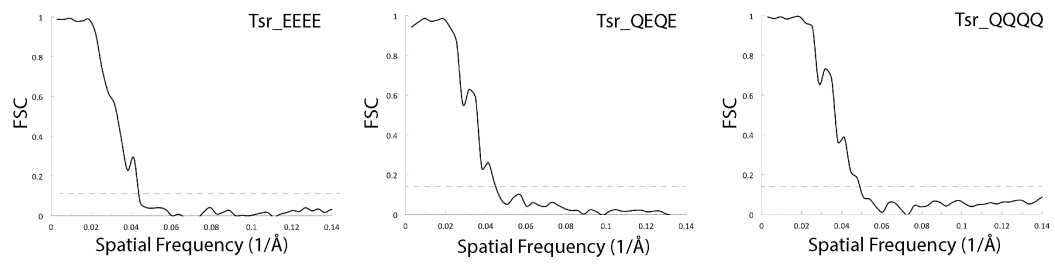

B

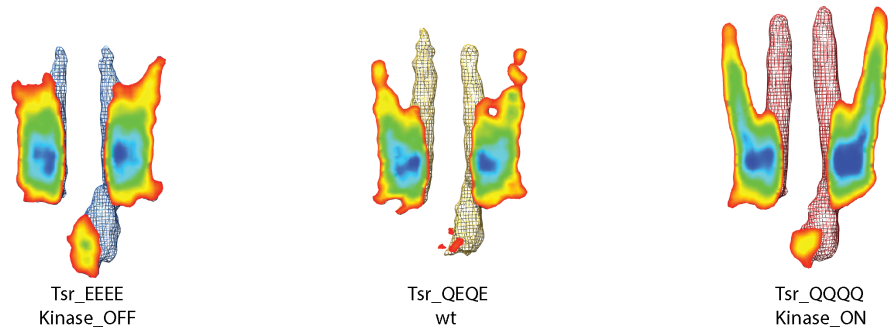

Supplement: FIG S4 [file mBio.00973-19-sf004.pdf]

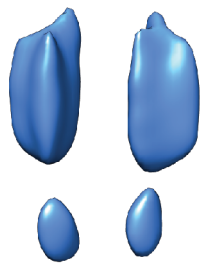

Tsr\_EEEE  
kinase\_OFF

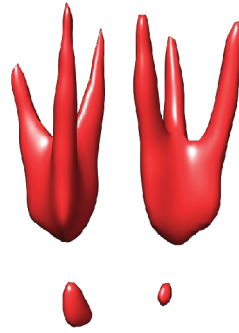

Tsr\_QQQQ  
kinase\_ON

Supplement: FIG S5 [file mBio.00973-19-sf005.pdf]

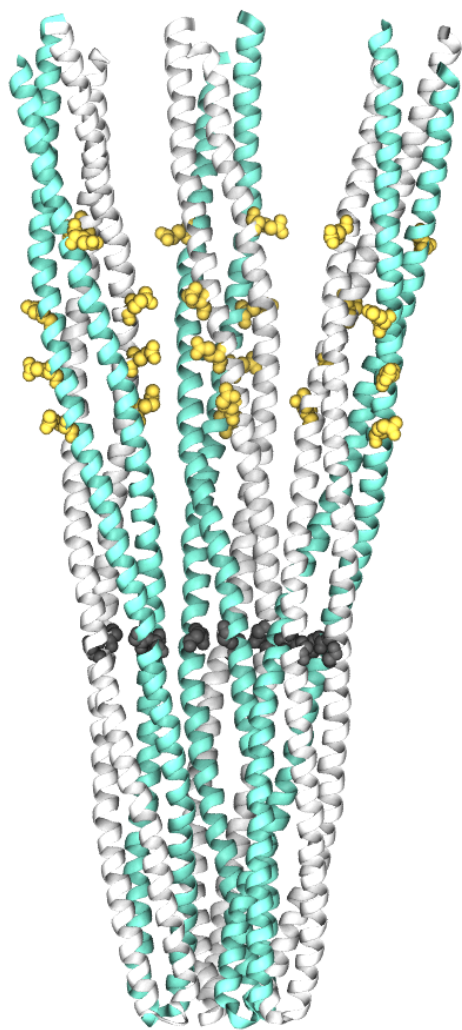

Supplement: FIG S6 [file mBio.00973-19-sf006.pdf]

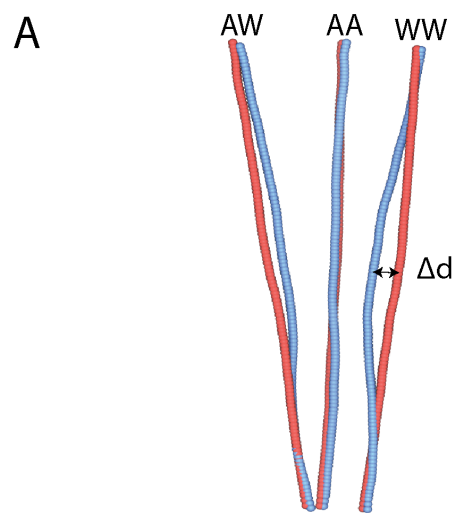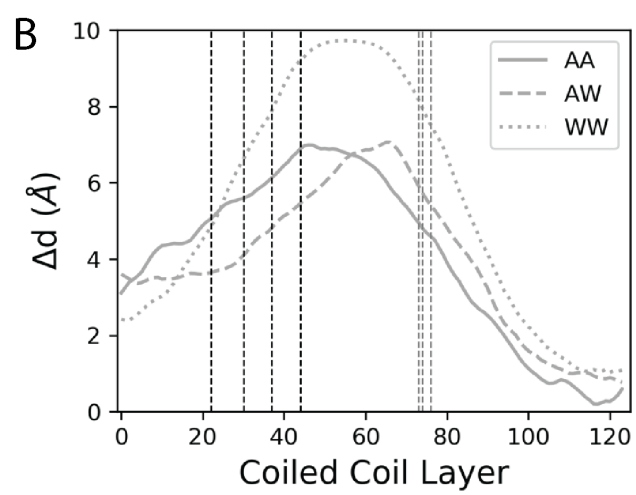

Supplement: FIG S7 [file mBio.00973-19-sf007.pdf]
