# Supplementary material for: In Situ Conformational Changes of the Escherichia coli Serine Chemoreceptor in Different Signaling States
Source: mBio. 2019 Jul 2;10(4):e00973-19. doi: 10.1128/mBio.00973-19 (PMC6606802; doi:10.1128/mBio.00973-19)
Supplement: TABLE S1 [file mBio.00973-19-st001.pdf]

**Strains**	**Relevant genotype**	**Ref**
UU2981	(flgM)Δ494 tsr-QQQQE (tar-cheB)Δ4346Δaer1(trg)Δ4543	This work
UU2982	(flgM)Δ494 (tar-cheB)Δ4346 Δaer-1(trg)Δ4543	This work
UU2983	flgM)Δ494 tsr-EEEEE (tar-cheB)Δ4346 Δaer-1(trg)Δ4543	This work
