# Supplementary material for: In Situ Conformational Changes of the Escherichia coli Serine Chemoreceptor in Different Signaling States
Source: mBio. 2019 Jul 2;10(4):e00973-19. doi: 10.1128/mBio.00973-19 (PMC6606802; doi:10.1128/mBio.00973-19)
Supplement: TABLE S2 [file mBio.00973-19-st002.pdf]

	Tsr_QQQQ	Tsr_QEQE	Tsr_EEEE
Receptor hexagon	1251	1011	1118
Signaling core unit	1977	2813	3148
Receptor trimer	3613	5686	6017
